# Supplementary material for: Integrated Proteomics and Metabolomics Link Acne to the Action Mechanisms of Cryptotanshinone Intervention
Source: Front Pharmacol. 2021 Sep 1;12:700696. doi: 10.3389/fphar.2021.700696 (PMC8440807; doi:10.3389/fphar.2021.700696)
Supplement: Supplementary file 1 [file DataSheet1.ZIP › Supplementary table 1-10 and material 1-3/Material 3-Metlib-MSMS/NEG-Metlib-MSMS/Metlib-MSMS/M242T340_forward/0.746,Cytidine,(M-H)-.pdf]

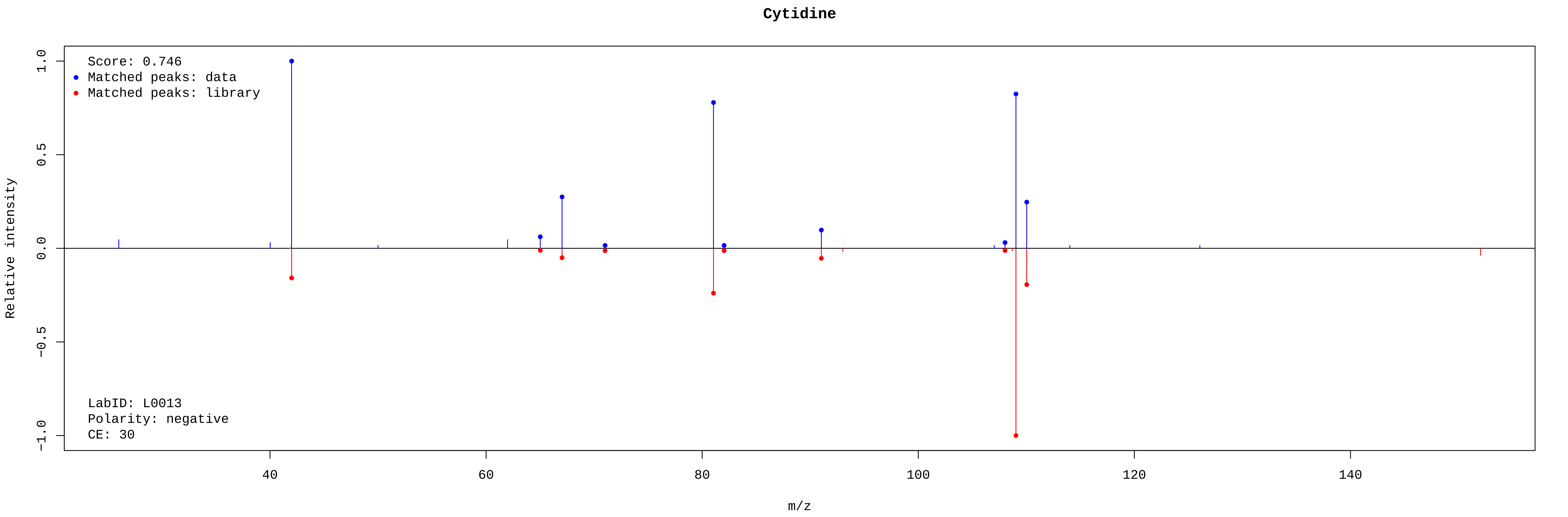

Cytidine
Score: 0.746
Matched peaks: data
Matched peaks: library
LabID: L0013
Polarity: negative
CE: 30
Relative intensity
1.0
0.5
0.0
-0.5
-1.0
m/z
40
60
80
100
120
140
